# Redox Regulation in Cancer Cells during Metastasis

**DOI:** 10.1158/2159-8290.CD-21-0558

**Published:** 2021-10-14

**Authors:** Alpaslan Tasdogan, Jessalyn M. Ubellacker, Sean J. Morrison

**Affiliations:** 1 Children's Research Institute and Department of Pediatrics, The University of Texas Southwestern Medical Center, Dallas, Texas.; 2 Howard Hughes Medical Institute, The University of Texas Southwestern Medical Center, Dallas, Texas.

## Abstract

Metastasis is an inefficient process in which the vast majority of cancer cells are fated to die, partly because they experience oxidative stress. Metastasizing cancer cells migrate through diverse environments that differ dramatically from their tumor of origin, leading to redox imbalances. The rare metastasizing cells that survive undergo reversible metabolic changes that confer oxidative stress resistance. We review the changes in redox regulation that cancer cells undergo during metastasis. By better understanding these mechanisms, it may be possible to develop pro-oxidant therapies that block disease progression by exacerbating oxidative stress in cancer cells.

**Significance:**

Oxidative stress often limits cancer cell survival during metastasis, raising the possibility of inhibiting cancer progression with pro-oxidant therapies. This is the opposite strategy of treating patients with antioxidants, an approach that worsened outcomes in large clinical trials.

## Introduction

Metastasis is the leading cause of death in patients with cancer because disseminated disease is no longer curable by surgery and is often therapy-resistant (1). Metastasis requires cancer cells to delaminate from their tumor of origin, invade the surrounding tissue, then migrate through tissue, blood, and/or lymph to new sites, all while surviving diverse and changing environments (2). Very few cancer cells survive this process, and many that do are unable to proliferate or persist in metastatic sites (3–6).

Cancer cells must be plastic to survive metastasis (7, 8). Genetic heterogeneity increases with disease progression (9), contributing to therapy resistance (10). Whole-genome duplications, chromosomal rearrangements, and chromosomal instability contribute to the increase in genetic heterogeneity (9, 11, 12). The genetic changes do not appear to confer metastatic competence, but rather arise by chance within primary tumors and are positively or negatively selected during metastasis (8, 11, 13). For example, copy-number changes in MYC (14) or MAPK pathway components (15) can enhance survival during metastasis. Recurrent coding sequence mutations have generally not been observed to arise during metastasis (11, 15–17), suggesting that there are not specific metastasis suppressor mutations. Rather, cancer cells undergo epigenetic (18, 19), transcriptional (7, 20–22), and metabolic (23–25) changes during metastasis. These reversible sources of heterogeneity conspire with genetic heterogeneity to confer fitness upon rare cells to survive and grow in metastatic sites.

Multiple factors contribute to the death of cancer cells during metastasis, including immune-mediated destruction (26, 27), growth factor deprivation (28), and diverse metabolic stresses (29). Redox stress is one important metabolic stress that limits the survival of cancer cells (24, 30). We review the role of redox regulation in metastasis and the metabolic adaptations that confer oxidative stress resistance.

## Metastasizing Cells Experience Oxidative Stress

**Figure 1.**
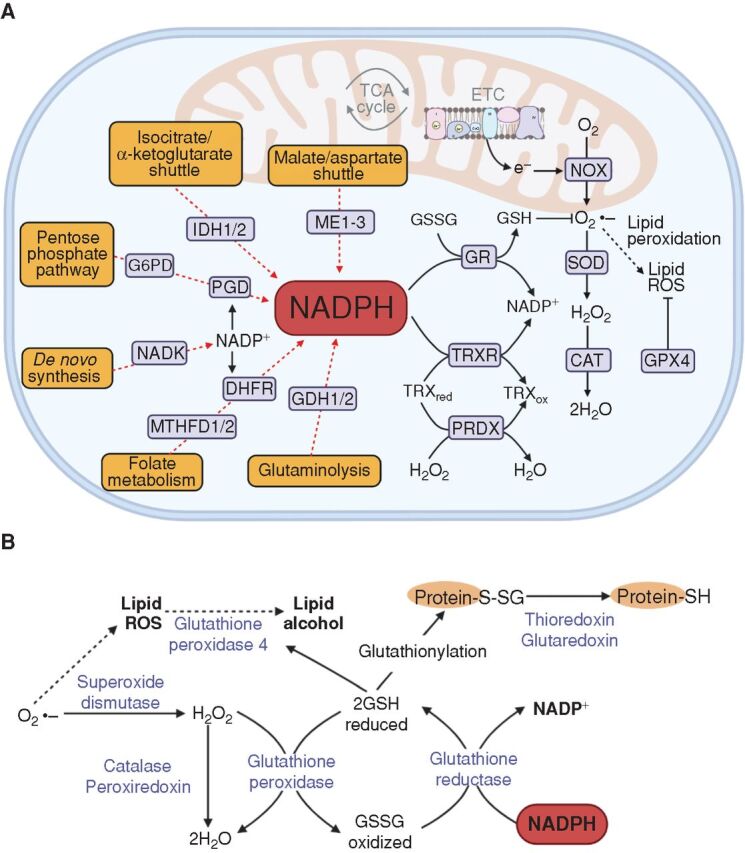
Metabolic pathways that generate NADPH are important sources of reducing equivalents for oxidative stress resistance. **A,** Glutathione (GSH) and thioredoxin (TRX_red_) are redox buffers that are used by antioxidant enzymes such as superoxide dismutase (SOD), peroxiredoxin (PRDX), and glutathione peroxidase 4 (GPX4) to neutralize ROS, including O_2_^−^, H_2_O_2_, and lipid ROS. The reduced forms of GSH and TRX can then be regenerated from the oxidized forms [glutathione disulfide (GSSG); TRX_ox_] by glutathione reductase (GR) or thioredoxin reductase (TRXR), which obtain reducing equivalents from NADPH. NADP^+^ is generated *de novo* from NAD^+^ by NAD kinase (NADK; ref. 167). NADP^+^ is then reduced to NADPH by the pentose phosphate pathway, the folate pathway, malic enzyme (ME1, 2, or 3), glutamate dehydrogenase (GDH1/2), or isocitrate dehydrogenase (IDH1/2; ref. 86). Other abbreviations in this panel include electron transport chain (ETC), glucose-6-phosphate dehydrogenase (G6PD), phosphogluconate dehydrogenase (PGD), dihydrofolate reductase (DHFR), methylenetetrahydrofolate dehydrogenase 1/2 (MTHFD1/2), NADPH oxidase (NOX), superoxide dismutase (SOD), and catalase (CAT). **B,** Schematic of reactions in which antioxidant enzymes transfer reducing equivalents between NADPH, GSH, and ROS.

Cancer cells experience oxidative stress during certain critical phases of their evolution and progression. The mechanisms that cause cancer cells to experience oxidative stress are poorly understood but likely include hyperactivation of anabolic pathways (31, 32), increased mitochondrial function (33), malfunction of the electron transport chain as a result of mitochondrial DNA mutations (34, 35), and oncogenic pathway activation (36–38). As a consequence, cancer cells are often more dependent than normal cells upon cellular antioxidants including glutathione (39, 40), thioredoxin (39), antioxidant enzymes (e.g., glutathione peroxidase, ref. 41; catalase, ref. 42; and superoxide dismutase, refs. 43, 44), and their transcriptional regulators, such as Nrf2 (45, 46) and BACH1 (ref. 47; Fig. 1A). Glutathione is an abundant redox buffer that is present mainly in the reduced form within cells. It opposes the development of oxidative stress by neutralizing (reducing) reactive oxygen species (ROS) including oxygen free radicals, peroxides, and lipid peroxides, as well as by glutathionylating thiol groups on proteins to protect them from oxidation (Fig. 1B). Glutathione can be regenerated from its oxidized form, glutathione disulfide, by glutathione reductase, using a reducing equivalent from NADPH. Consequently, metabolic pathways that generate NADPH from NADP^+^ are important sources of reducing equivalents for oxidative stress resistance (ref. 48; Fig. 1A).

Cancer cells that survive the oxidative stress they experience during transformation (39) are able to bring oxidative stress under control, allowing the activation of anabolic pathways to drive tumor growth (31). However, when cells in primary tumors detach from extracellular matrix during invasion, they experience changes in signaling pathway activation and metabolism that again increase oxidative stress (49–51). There is evidence that cancer cells either proliferate or invade surrounding tissues but generally do not do both at the same time (52, 53), raising the possibility that invasion requires cells to downregulate anabolic pathways.

Oxidative stress likely increases further when metastasizing cancer cells enter the blood (24, 54–57), which has among the highest levels of oxidants in the body, including oxygen and iron. Oxidative stress limits the survival of cancer cells during metastasis (24). Treatment of mice with antioxidants increases the frequency of circulating cancer cells in the blood and metastatic disease burden (24, 30, 47, 58, 59). This has been observed in multiple cancers, in patient-derived xenografts growing in immunocompromised mice as well as in mouse cancers growing in syngeneic immunocompetent mice. Consistent with this, cancer cells undergo metabolic changes during invasion and metastasis that would be expected to reduce the generation of ROS (60–65).

Nascent metastatic nodules continue to exhibit signs of oxidative stress, including increased ROS levels and low ratios of glutathione to oxidized glutathione and NADPH to NADP^+^ (24), although the degree of oxidative stress differs among sites of metastasis (66). Oxidative stress is likely to slow the ability of metastatic cells, at least in some sites, to fully reactivate the anabolic pathways required for tumor growth, even after they have extravasated from the blood. For example, lipogenesis requires reducing equivalents from NADPH; inhibiting acetyl-CoA carboxylase decreases NADPH consumption by fatty acid synthesis and preserves NADPH for other cellular processes (67). Cancer cells shut down anabolic pathways during metastasis to conserve reducing equivalents to manage oxidative stress. Indeed, it is conceivable that dormancy in metastatic cells is sometimes caused by a prolonged failure to fully bring oxidative stress under control, leading to prolonged quiescence. Nonetheless, once metastatic tumors have grown beyond a few millimeters in diameter, cancer cells likely have undergone the adaptations needed to control oxidative stress, allowing broad activation of anabolic pathways.

Dietary supplementation with antioxidants has been proposed to provide health benefits, including suppressing the development of cancer by reducing ROS levels (68). Consequently, many clinical trials have been performed to test whether dietary supplementation with antioxidants can suppress the development of cancer. However, dietary antioxidants have consistently failed to reduce cancer incidence or cancer-related deaths in human clinical trials (69). Consistent with the data from experimental models, dietary supplementation with antioxidants in humans tended to increase cancer incidence and cancer-related deaths (70–73). The data thus suggest that antioxidants generally promote the development and progression of cancer in both animal models and in humans.

Although oxidative stress commonly limits the survival of cancer cells during transformation and metastasis, ROS also promotes cancer progression in certain contexts (74). ROS can cause DNA damage, contributing to the formation of oncogenic mutations, and can serve as progrowth signaling molecules (33). Genetic changes that increase the generation of ROS can promote cancer progression, and treatment with antioxidants has sometimes been observed to inhibit metastasis (75–79). For example, inhibition of TIGAR, an enzyme that promotes the entry of glucose into the pentose phosphate pathway, increases ROS levels in pancreatic ductal adenocarcinoma, leading to increased migration, invasion, and metastasis (80). One possibility is that modest increases in ROS levels can promote the activation of signaling pathways that are adaptive for cancer cells (33), particularly in early-stage cancers, while the higher ROS levels observed in metastasizing cancer cells are toxic. Another possibility is that different types of ROS have different effects on cancer cells. For example, hydrogen peroxide created by mitochondrial ROS might promote metastasis (81), whereas lipid peroxides created by membrane lipid oxidation might undermine survival during metastasis (55).

There may also be differences among cancers or model systems, in which oxidative stress limits disease progression in certain cancers while promoting disease progression in others. It is conceivable that mouse models of cancer tend to have lower ROS levels than human cancers due to lower mutation burdens. Cancer cell lines may have been selected for the capacity to withstand oxidative stress as a result of being propagated in culture. These will be important possibilities to consider as the field dissects the role of ROS and oxidative stress in cancer progression.

## Mechanisms of Oxidative Stress Resistance During Metastasis

There are heritable, stable, and cell-intrinsic differences among cancers in their metastatic potential based on metabolic and transcriptional differences, including those that confer oxidative stress resistance (54, 82, 83). There is also heterogeneity among cancer cells within the same tumor that influences metastatic potential (54, 84, 85). For example, melanoma cells within hypoxic regions of primary tumors express higher levels of the lactate transporter MCT1, and higher levels of MCT1 confer oxidative stress resistance that increases survival in the blood (54). MCT1 seems to promote oxidative stress resistance by increasing lactate uptake, which decreases intracellular pH and the NAD^+^/NADH ratio. This promotes pentose phosphate pathway function, a major source of NADPH for oxidative stress resistance (86). Consistent with this, hypoxic cells within primary tumors exhibit transcriptional changes that appear to confer an oxidative stress–resistant phenotype that promotes the survival of metastasizing cells in the blood, increasing their potential to form metastatic tumors (87). Increased MCT1 expression may be one element of this phenotype.

*De novo* serine synthesis (88) and serine degradation (89) both yield NADPH and are used by cancer cells to manage oxidative stress, particularly during hypoxia. Although cancer cells that metastasize through the blood would not be expected to be hypoxic, these pathways nonetheless promote metastasis, potentially by acting in cancer cells within hypoxic environments (e.g., in lymph or after extravasation into metastatic sites). Inhibition of either phosphoglycerate dehydrogenase, an enzyme involved in serine synthesis, or serine hydroxymethyltransferase, an enzyme involved in serine degradation, increases ROS levels and reduces the formation of metastatic tumors (88, 89). Serine biosynthesis also preferentially promotes the growth of metastatic tumors as compared with primary tumors by promoting mTORC1 signaling (90). It is not clear whether the change in mTORC1 signaling contributes to the change in ROS levels. ROS also induces the expression of β-globin, the oxygen-binding protein best known for its function in erythrocytes, in circulating breast cancer cells (57). This appears to protect the cancer cells from oxidative stress, perhaps by scavenging ROS.

There is genetic evidence that some cancers, including melanoma and lung cancer, give rise to polyclonal metastases (91, 92). There are likely multiple cellular mechanisms that contribute to the formation of polyclonal metastases, including metastasis-to-metastasis spread (93). Another mechanism that may contribute to polyclonal metastasis is that some circulating cancer cells move through the blood in clusters. Clustering can occur among cancer cells or between cancer cells and neutrophils. In both cases it increases cancer cell survival and their ability to form metastatic tumors as compared with single circulating cancer cells (94–96). Clustering may promote the survival of cancer cells in the blood partly by reducing their exposure to oxygen, reducing the production of mitochondrial ROS (97). E-cadherin expression by metastasizing cells also promotes survival by limiting oxidative stress (98). It is tempting to speculate that E-cadherin acts by promoting cell–cell interaction, although E-cadherin deletion does not reduce the fraction of cancer cells that are present in cell clusters.

**Figure 2.**
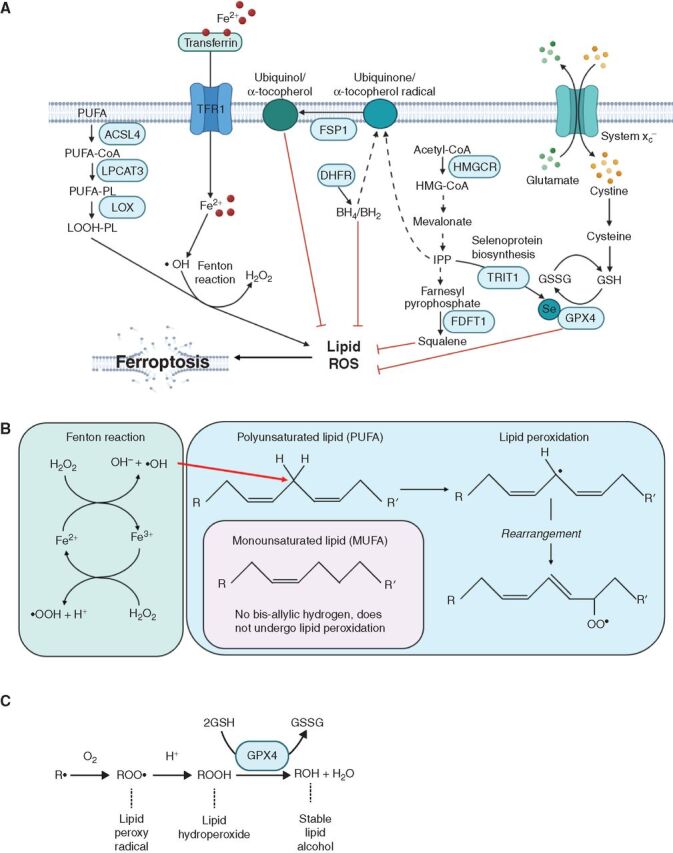
The regulation of ferroptosis. **A,** Lipid ROS, including lipid peroxides, arise as a result of the oxidation of polyunsaturated fatty acids (PUFA), driven by Fenton reactions in which redox active iron generates hydroxyl radicals (•OH). These PUFAs are present in membrane phospholipids (PL). Cells have multiple antioxidant defenses that oppose the accumulation of lipid ROS including the selenocystine (Se) enzyme, glutathione peroxidase 4 (GPX4), and the reducing agents squalene (100), tetrahydrobiopterin (BH_4_; ref. 105), and ubiquinol/α-tocopheral. Abbreviations include transferrin receptor protein 1 (TFR1), acyl-CoA synthetase long-chain family member 4 (ACSL4), lysophosphatidylcholine acyltransferase 3 (LPCAT3), lysyl oxidase (LOX), six-transmembrane epithelial antigen of prostate 3 (STEAP3), divalent metal transporter 1 (DMT1), ferroptosis suppressor protein 1 (FSP1; refs. 168, 169), dihydrofolate reductase (DHFR), 3-Hydroxy-3-Methylglutaryl-CoA Reductase (HMGCR), TRNA Isopentenyltransferase 1 (TRIT1), glutathione (GSH), glutathione disulfide (GSSG), farnesyl-diphosphate farnesyltransferase 1 (FDFT1). **B,** Schematic of reactions in which iron generates hydroxyl radicals (•OH) that react with bis-allylic hydrogens in PUFAs to generate lipid ROS (99). **C,** Generation of stable lipid alcohols from lipid ROS by GPX4.

Oxidative stress kills metastasizing cancer cells by inducing ferroptosis (55, 56), a form of cell death marked by lipid oxidation (Fig. 2A; ref. 99). During ferroptosis, polyunsaturated fatty acids (PUFA) in membrane phospholipids are oxidized by redox-active iron. The resulting lipid peroxides can be scavenged by dietary antioxidants such as vitamin E or by certain cellular antioxidant defenses, such as GPX4 (100–102); however, accumulation of the lipid peroxides can overwhelm the cellular antioxidant defenses, leading to the induction of ferroptosis. At least in melanoma, ferroptosis does not appear to limit the growth of primary cutaneous tumors, in which little oxidative stress is evident, but does limit the survival of metastasizing cells (55). Circulating melanoma cells attempt to manage lipid oxidation by increasing the transcription of transferrin, which reduces intracellular iron levels and lipid peroxidation (56), and by increasing the incorporation of monounsaturated fatty acids (MUFA) into membrane lipids to displace PUFAs (55). Ferroptosis sensitivity marks a therapy-resistant cell state that is observed across several cancers, including melanoma, and that involves the increased synthesis of PUFAs (103), including polyunsaturated ether phospholipids (104). This raises the possibility that many cancers may become more sensitive to ferroptosis during metastasis and that disease progression could be inhibited by interventions that increase lipid peroxidation (85, 103–105).

The susceptibility of metastasizing cancer cells to ferroptosis appears to be influenced by both cell-autonomous lipid metabolism and by lipids taken up from the environment. Fatty acid transporters, including CD36, tend to be more highly expressed by cancer cells as compared with normal cells and promote metastasis or poor prognosis in multiple cancers (106, 107). Stearoyl-CoA desaturase (SCD1) is involved in the conversion of saturated to MUFAs in melanoma cells. Melanomas that are high for the Microphthalmia-associated transcription factor (MITF), which promotes aggressive proliferation but suppresses invasion (108), are dependent upon SCD1, perhaps to sustain membrane lipid biosynthesis (85, 109, 110). In contrast, melanomas that are low for MITF and less proliferative but more invasive are less dependent upon SCD1 (85). One possibility is that these MITF^lo^ melanomas become more dependent upon MUFAs taken up from their environment during metastasis (55) because there is less SCD1-mediated production of MUFAs cell-intrinsically.

The literature on the effects of a high-fat diet on cancer is mixed (111). Some studies found that high-fat diets (112, 113) or dietary supplementation with palmitic acid, a saturated fatty acid (106), can promote metastasis. Other studies found that ketogenic high-fat diets can reduce metastatic disease burden, partly by increasing oxidative stress in cancer cells (114, 115). One possibility is that variability in outcomes among studies reflects differences in the PUFA or MUFA content of the diets that were administered. Many factors likely contribute to these differences in outcomes, including differences among high-fat diets in fatty acid, protein, and carbohydrate composition. In addition to the effects of fatty acids on redox homeostasis, fatty acids also play critical roles in membrane biosynthesis and energy metabolism that have effects on cancer progression independent of the effects on redox status (85, 116, 117).

## Metastasis Through Lymphatics

Many cancers, including epithelial cancers and melanomas, form metastases in draining lymph nodes prior to forming metastases at distant sites (118–121). Genetic studies in human and mouse cancers have shown that regional lymph node metastases can give rise to distant metastases (91–93, 122). In mouse models, cancer cells in lymph nodes are capable of metastasizing to distant sites through the blood (123–125). However, some distant metastases arise from clones that differ from those in lymph nodes. In these instances, it is possible the metastatic cells entered the blood directly from primary tumors, or transited through lymphatics without forming lymph node tumors (92, 122). Obviously, it is also possible that they formed lymph node tumors that were neither detected nor sampled for analysis.

Lymphatics promote the migration and survival of cancer cells. Some cancers form more tumors after intralymphatic injection as compared with intravenous injection (55, 126). VEGFC and various chemokines promote the migration of cancer cells into lymphatic vessels, facilitating metastatic spread (127–131). When VEGFC is overexpressed in mouse lungs, it increases lymphatic vessel density, increasing the spread of cancer cells from the lung to other organs (131). The capacity to oxidize fatty acids promotes the survival of cancer cells in lymphatics (132) and their formation of metastatic tumors (106). Consistent with this, fatty acid oxidation promotes oxidative stress resistance and metastatic potential in colorectal cancer cells (133).

Melanoma cells that metastasize through lymph are metabolically different from cells that metastasize through blood (55). Melanoma cells in lymph experience less oxidative stress and form more metastases than melanoma cells in the blood (55). One of the ways in which lymph protects from ferroptosis is by having high levels of the MUFA oleic acid, which protects cells from lipid oxidation by reducing the abundance of PUFAs in membranes. PUFAs, but not MUFAs, are oxidized during ferroptosis due to the bis-allylic hydrogens they contain (Fig. 2B and C; ref. 99). Compared with the blood, lymph also contains lower concentrations of oxygen and iron, oxidants that contribute to ferroptosis (55). These observations suggest that melanoma cells tend to metastasize initially through lymphatics because lymph protects them from oxidative stress. Moreover, while in lymph, cancer cells increase MUFA incorporation into phospholipids, reducing their susceptibility to ferroptosis when they subsequently enter the blood.

## Mitochondrial Function as a Determinant of Metastasis

Mitochondrial function has been studied only to a limited extent in cancer cells during metastasis, leaving many questions unanswered. One of the key impediments is that circulating cancer cells are rare, making it difficult to obtain enough cells for many assays. Nonetheless, mitochondria are a major source of ROS in cells and there is increasing evidence that mitochondrial function reduces the survival of metastasizing cancer cells, at least partly by increasing ROS levels (134). Mitochondrial mass and mitochondrial membrane potential decline in circulating melanoma cells in the blood as compared with the primary tumors from which they arise (24). One possibility is that these changes reflect decreased mitochondrial function in an effort to manage the production of mitochondrial ROS. However, flow cytometric measurements of mitochondrial membrane potential do not always correlate with mitochondrial or electron chain function (135). Lung cancer cell lines with metastatic potential have lower mitochondrial membrane potential and reduced mitochondrial function as compared with nonmetastatic lung cancer cell lines (136). PGC1α, a transcription factor that promotes mitochondrial biogenesis, seems to promote invasion and metastasis in some contexts (76) while inhibiting metastasis in others, including in melanoma (84, 137). Melanoma cells in primary tumors are heterogeneous for PGC1α expression, with PGC1α^lo^ cells exhibiting increased metastatic potential, again consistent with the idea that reduced mitochondrial function promotes metastasis (84). However, there are many mechanisms downstream of PGC1α that appear to contribute to its effects on metastasis, including mechanisms independent of mitochondrial function (76, 84, 137). Additional studies of mitochondrial function during metastasis are required.

Metabolic pathways associated with mitochondrial function influence metastatic potential. For example, increased asparagine availability, either from the diet or from biosynthesis, promotes metastasis (138). Asparagine is synthesized from aspartate, and aspartate synthesis depends on electron transport chain function (139–141). This raises the possibility that asparagine is limiting in metastasizing cancer cells because mitochondrial function is limited in an effort to manage oxidative stress (136).

## Pro-Oxidant Therapies

**Figure 3.**
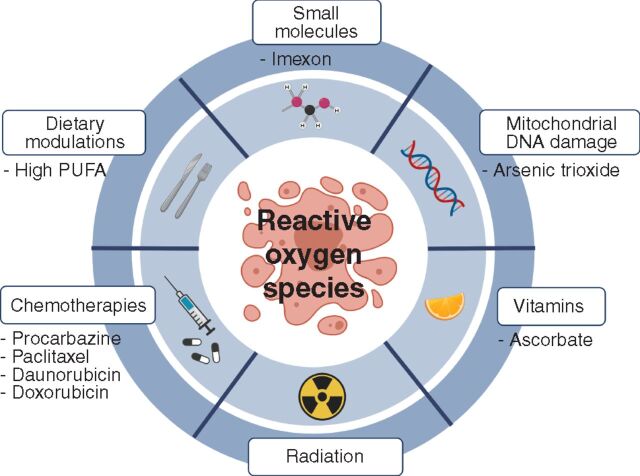
Potential pro-oxidant therapies. It may be possible to inhibit the metastasis or progression of some cancers using pro-oxidant therapies that exacerbate the oxidative stress experienced by cancer cells.

The studies above suggest that cancer progression might be inhibited with pro-oxidant therapies that exacerbate oxidative stress in cancer cells or block the metabolic adaptations that confer oxidative stress resistance (ref. 142; Fig. 3). The anticancer activity of radiation reflects, in part, the formation of hydroxyl radicals that attack DNA (143). Widely used chemotherapies, including procarbazine, paclitaxel, daunorubicin, and doxorubicin, kill cancer cells partly by promoting oxidative stress (144–146). Many small-molecule drugs with direct or indirect pro-oxidant effects have been tested in clinical trials for a wide range of cancers (147), and new strategies for developing prooxidant small molecules are being explored (148, 149). For example, Imexon is a small molecule that binds to thiols, depleting glutathione and increasing ROS levels, which has been tested for activity against non-Hodgkin lymphoma (150). Arsenic trioxide is used for the treatment of acute promyelocytic leukemia and may act partly by impairing electron transport chain function, leading to electron leakage and the generation of superoxide (151). These ROS-generating agents might damage mitochondrial DNA, which is more vulnerable to ROS than nuclear DNA (152), further increasing the generation of ROS as a result of defects in electron transport chain function (153). While a number of effective anticancer therapies have pro-oxidant effects, it is uncertain to what extent their anticancer activities reflect these pro-oxidant activities as compared with other activities independent of ROS.

Ascorbate (vitamin C) is generally considered an antioxidant, but it exists in oxidized and reduced forms and when it is infused intravenously it selectively kills cancer cells by acting as a pro-oxidant (154). This is because the superphysiologic levels of ascorbate that can be achieved by intravenous infusion lead to the uptake of the fully oxidized form of ascorbate, dehydroascorbate, via the GLUT1 transporter, which is highly expressed in cancer cells with MAPK pathway activation. Once taken up by the cancer cells, dehydroascorbate is reduced back to ascorbate, inducing oxidative stress by consuming reducing equivalents. Ascorbate also alters the activity of epigenetic enzymes, such as TET2, which use ascorbate as a cofactor (155, 156). Building on the original studies by Linus Pauling that reported prolonged survival in patients with cancer administered high-dose intravenous ascorbate (157), the recent work demonstrating the pro-oxidant and epigenetic effects of high-dose ascorbate has led to a number of clinical trials testing activity against a wide range of cancers (158).

Dietary interventions could also have pro-oxidant effects. Ketogenic diets may suppress metastasis partly by increasing oxidative stress in cancer cells (114). Ketogenic diets are designed to minimize dietary carbohydrates, reducing blood glucose and insulin levels (159). However, ketogenic diets also increase dietary fat, commonly increasing PUFA levels. Increased incorporation of PUFAs into membrane phospholipids renders cancer cells more susceptible to the accumulation of lipid ROS and ferroptosis (160). This raises the possibility that ketogenic diets may exert anticancer effects partly by altering lipid metabolism (161) or by increasing PUFA levels in the membranes of cancer cells (162). Nonetheless, it remains to be tested whether a high PUFA diet or other approaches to promote PUFA incorporation into cancer cells could inhibit disease progression.

## Future Directions

New technical approaches to study metastasis, including whole-body imaging of metastasis patterns (163), improved techniques for the isolation of circulating cancer cells from patients (164), screens to identify gene products that modulate metastasis (165, 166), and lineage tracing of bar-coded cancer cells to trace routes of metastasis (83), are accelerating progress.

In at least some cancers, metastasizing cells appear to experience unusually high levels of oxidative stress, raising the possibility that these cells might be particularly sensitive to pro-oxidant therapies. It is an open question whether such therapies could prevent disease progression in patients with high-risk primary or regionally metastatic lesions. Nonetheless, this merits deeper study in preclinical models. Beyond this big-picture question, there are a number of pressing biological questions central to understanding redox regulation during metastasis:

- Does oxidative stress limit the survival of metastasizing cells from all cancers or only certain cancers?
- What causes the oxidative stress experienced by metastasizing cells?
- Are anabolic pathways downregulated in metastasizing cells to preserve reducing equivalents? Does this sometimes lead to dormancy in metastatic cells?
- How is mitochondrial function modulated in metastasizing cancer cells as compared with the primary tumors from which they arise?
- Do micrometastases continue to experience oxidative stress? For how long?
- To what extent do interactions with immune and stromal cells influence oxidative stress in cancer cells?
- Do differences in oxidative stress among distinct metastatic sites influence organotropism?
